# Pulmonary, cardiac and renal distribution of ACE2, furin, TMPRSS2 and ADAM17 in rats with heart failure: Potential implication for COVID‐19 disease

**DOI:** 10.1111/jcmm.16310

**Published:** 2021-03-04

**Authors:** Emad E. Khoury, Yara Knaney, Ahmad Fokra, Safa Kinaneh, Zaher Azzam, Samuel N. Heyman, Zaid Abassi

**Affiliations:** ^1^ Department of Physiology and Biophysics Rappaport Faculty of Medicine Technion‐Israel Institute of Technology Haifa Israel; ^2^ Department of Internal Medicine Rambam Medical Center Haifa Israel; ^3^ Department of Medicine Hadassah Hebrew University Hospital Jerusalem Israel; ^4^ Laboratory Medicine Rambam Medical Center Haifa Israel

**Keywords:** ADAM17, angiotensin converting enzyme 2, furin, heart, heart failure, kidney, lung, TMPRSS2

## Abstract

Congestive heart failure (CHF) is often associated with kidney and pulmonary dysfunction. Activation of the renin‐angiotensin‐aldosterone system (RAAS) contributes to avid sodium retention, cardiac hypertrophy and oedema formation, including lung congestion. While the status of the classic components of RAAS such as renin, angiotensin converting enzyme (ACE), angiotensin II (Ang II) and angiotensin II receptor AT‐1 is well studied in CHF, the expression of angiotensin converting enzyme‐2 (ACE2), a key enzyme of angiotensin 1‐7 (Ang 1‐7) generation in the pulmonary, cardiac and renal systems has not been studied thoroughly in this clinical setting. This issue is of a special interest as Ang 1‐7 counterbalance the vasoconstrictory, pro‐inflammatory and pro‐proliferative actions of Ang II. Furthermore, CHF predisposes to COVID‐19 disease severity, while ACE2 also serves as the binding domain of SARS‐CoV‐2 in human host‐cells, and acts in concert with furin, an important enzyme in the synthesis of BNP in CHF, in permeating viral functionality along TMPRSST2. ADAM17 governs ACE2 shedding from cell membranes. Therefore, the present study was designed to investigate the expression of ACE2, furin, TMPRSS2 and ADAM17 in the lung, heart and kidneys of rats with CHF to understand the exaggerated susceptibility of clinical CHF to COVID‐19 disease. Heart failure was induced in male Sprague Dawley rats by the creation of a surgical aorto‐caval fistula. Sham‐operated rats served as controls. One week after surgery, the animals were subdivided into compensated and decompensated CHF according to urinary sodium excretion. Both groups and their controls were sacrificed, and their hearts, lungs and kidneys were harvested for assessment of tissue remodelling and ACE2, furin, TMPRSS2 and ADAM17 immunoreactivity, expression and immunohistochemical staining. ACE2 immunoreactivity and mRNA levels increased in pulmonary, cardiac and renal tissues of compensated, but not in decompensated CHF. Furin immunoreactivity was increased in both compensated and decompensated CHF in the pulmonary, cardiac tissues and renal cortex but not in the medulla. Interestingly, both the expression and abundance of pulmonary, cardiac and renal TMPRSS2 decreased in CHF in correlation with the severity of the disease. Pulmonary, cardiac and renal ADAM17 mRNA levels were also downregulated in decompensated CHF. Circulating furin levels increased in proportion to CHF severity, whereas plasma ACE2 remained unchanged. In summary, ACE2 and furin are overexpressed in the pulmonary, cardiac and renal tissues of compensated and to a lesser extent of decompensated CHF as compared with their sham controls. The increased expression of the ACE2 in heart failure may serve as a compensatory mechanism, counterbalancing the over‐activity of the deleterious isoform, ACE. Downregulated ADAM17 might enhance membranal ACE2 in COVID‐19 disease, whereas the suppression of TMPRSS2 in CHF argues against its involvement in the exaggerated susceptibility of CHF patients to SARS‐CoV2.

## 1 INTRODUCTION

COVID‐19 pandemic is associated with high morbidity and mortality among susceptible patients in the absence of an effective treatment.^1^ Yet, the pathogenesis of SARS‐CoV‐2‐induced injury to vital organs, including the lungs, heart and kidneys, is not fully understood, as is the propensity of aged patients with diabetes, hypertension, heart failure and respiratory diseases to develop severe illness.^1^, ^2^, ^3^, ^4^, ^5^, ^6^, ^7^, ^8^ The common denominator of these COVID‐19‐target organs is the high abundance of angiotensin converting enzyme 2 (ACE2) and activating enzymes TMPRSS2 (Transmembrane serine protease II)/furin, which are exploited by the SARS‐CoV‐2 to bind and invade target organ cells.^9^, ^10^, ^11^, ^12^, ^13^ ACE2, the binding site of the virus in human host‐cells, is a transcellular protein predominantly expressed in the heart, vasculature, kidneys, lungs, brain, intestine and testis.^4^, ^14^, ^15^, ^16^ ACE2 has a physiologic role by cleaving Angiotensin (Ang) I into Ang 1‐9, which can be further converted to Ang 1‐7 by ACE.^14^, ^15^, ^17^ It also generates Ang 1‐7 directly from Ang II.^14^, ^15^, ^17^ Ang II and Ang 1‐7 have prominent opposing physiologic impact on the regulation of microcirculation, blood pressure, inflammation, tissue remodelling and sodium/water homeostasis, and their balanced generation and activity are tightly controlled.^14^, ^15^, ^16^, ^17^ While Ang II promotes vasoconstriction, inflammation, fibrosis and sodium retention, Ang 1‐7 exerts vasodilation, suppresses inflammation and fibrosis, and promotes natriuresis. Interestingly, both clinical and experimental heart failure are associated with enhancement of cardiac ACE2 and Ang 1‐7 generation,^8^, ^14^, ^16^, ^18^, ^19^, ^20^, ^21^, ^22^ a phenomenon that may represent a counterbalancing compensatory response to Ang‐II‐induced vasoconstriction, cardiac remodelling and sodium retention.

The clinical presentation of patients with SARS‐CoV‐2 infection has stirred the interest in Ang II‐Ang 1‐7 balance, since the binding of SARS‐CoV‐2 spike protein to ACE2 was found to downregulate this organ‐protective enzyme and decreasing Ang 1‐7 production.^4^, ^14^, ^23^ It is tempting to assume that this undesired Ang 1‐7 / Ang II imbalance may underline many of the deleterious effects SARS‐CoV‐2 on the pulmonary, cardiac, renal and vascular systems, especially in patients with chronic underlying diseases.^4^, ^23^ Furthermore, blockade of the RAAS system by ACE inhibitors (ACEi) and angiotensin receptor blockers (ARBs) upregulates the expression of ACE2 in patients predisposed to severe SARS‐CoV‐2 infection, such as individuals with heart failure, already displaying abundant ACE2 in their COVID‐19‐target organs, thus potentially sensitizing patients on these medications to the virus.^21^, ^24^, ^25^ The mechanisms underlying the high susceptibility of CHF patients to COVID‐19 remain largely unknown. Therefore, alterations in ACE2 expression in patients with cardiovascular diseases should be taken into account when assessing their risk, morbidity and mortality related to the viral infection. Unfortunately, the status of ACE2 has mostly been determined in the circulation, limited to ACE2 peptides detached from cell membranes.^4^, ^26^, ^27^ There is limited post‐mortem data regarding its expression on cell membranes in patients with COVID‐19 infection, other than in cardiac tissues of patients with CHF,^8^ and its expression in pulmonary and renal tissues is largely unknown. Moreover, the status of other principal participants in viral attachment, such as furin, has not been studied thoroughly in target organs in patients prone to severe COVID‐19 infection, including those with CHF. Furin activates more than 150 substrates of mammalian, viral and bacterial origin, cleaving these substrates at basic residues with the typical recognition motif R‐Xn‐K/R‐R, hence given the name PACE (paired basic amino acid cleaving enzyme). In this context, genomic characterization of SARS‐CoV‐2 revealed that furin cleaves the S spike of the viral envelope glycoproteins, enhancing its fusion to cellular membrane.^28^, ^29^, ^30^ Noteworthy, furin is ubiquitously expressed in a broad range of cells within COVID‐19‐target organs, such as the lung, heart, gut and nasal mucosa,^31^, ^32^ where the mature and active form is present in the Golgi network and can be further transported to the cell membrane and back through the endosomal pathway.^31^ Additionally, furin can be cleaved by an unknown mechanism and shed into the extracellular space.^33^ In sum, so far, information concerning the expression and distribution of ACE2 and furin in target organs of SARS‐CoV‐2 in individuals with CHF is, unfortunately, limited. The impact of CHF on the expression of TMPRSS2 and ADAM17, additional mediators involved in SARS‐CoV‐2 attachment,^34^, ^35^ and in ACE2 membranal detachment,^36^ respectively, is also unknown. Elucidating these issues, may provide new insights into the physiology of CHF and regarding the pathologic mechanisms and susceptibility of patients with CHF to COVID‐19 infection, and might eventually pave the way for a better assessment and treatment of such patients in general and while infected with SARS‐CoV‐2.

## 2 METHODS

### 2.1 Experimental groups

Studies were conducted on male Sprague Dawley rats weighing 300‐350 g. The animals were kept in a temperature‐controlled room and fed with standard rat chow containing 0.5% NaCl and tap water ad libitum. All experiments were approved by the Animal Care and Use Committee of the Technion‐Israel Institute of Technology.

### 2.2 Experimental model

Aorto‐caval fistula (ACF) was induced in these rats by surgically creation of an incision (approximately 1.2‐1.4 mm in length) between the abdominal aorta and inferior vena cava, distal to the origin of the renal arteries as described previously.^37^ The opening in the vena cava is then closed by a continuous suture. A matched group of sham‐operated rats served as controls. After surgery, rats were allowed to recover and placed in metabolic cages for one week. Seven days after the operation, rats with ACF were divided into two subgroups according to their daily absolute rate of sodium excretion (UNaV): rats with decompensated CHF (UNaV < 200 µEq/24 hours) and rats with compensated CHF (UNaV > 1200 µEq/24 hours).^37^

The three subgroups of animals: sham‐operated rats (n = 9), compensated (n = 9) and decompensated (n = 11) animals were anesthetized with Nembutal (60 mg/kg, i.p.), and their hearts, lungs and kidneys were washed via right ventricle perfusion with 120 mL phosphate buffered saline (0.01 mol/L PBS, pH 7.4) containing heparin (5 U/mL). The heart was removed and dissected, where the left atrium, left ventricle, right atrium and right ventricle were separated. The kidney was longitudinally cut into two halves, where one half was homogenized for WB analyses either as a whole kidney or separated cortex and medulla, the second half for RT‐PCR. The right lung was utilized for WB and the left one for RT‐PCR analysis.

### 2.3 RT‐qPCR

Total RNA was isolated from snap‐frozen tissue samples using TRIzol^®^ Reagent (Life Technologies), according to the manufacturer's instructions, and quantified by spectrophotometry using NanoDrop 2000. After oligo (dT)‐primed reverse transcription of 1000 ng total RNA, the resulting single‐stranded cDNA was used for PCR. PCR conditions were as follows: an initial denaturation step at 95°C for 3 minutes, 30 cycles of denaturation at 95°C for 30 seconds, and hybridization at 60°C for 30 seconds followed by elongation at 72°C for 1 minute. Finally, the PCR reaction was terminated by incubation at 72°C for 5 minutes. GAPDH was used as an internal standard. The following primers were used:

**Table:** 

ACE2	F(5′‐AGAAGTTGCTCAATATGCTG‐3′)
	R(5′‐ATCCATATTCCTTGATCCTACC‐3′);
Furin	F(5′‐AGGGGTAGGCTGACATCATCT‐3′),
	R(5′‐CCAGGGCACAGTGTTAGTTTG‐3′);
TMPRSS2	F(5′‐TCCAGGTTTACTCATCTCAG‐3′)
	R(5′‐CCCTTGGCTAGAATAAAAGC‐3′);
ADAM17	F(5′‐GAGGTAAAACCTGGTGAAAG‐3′);
	R(5′‐TATCAAGGCTAAATTGCTCC‐3′);
GAPDH	F(5′‐GTGCCAGCCTCGTCTCATAG‐3′);
	R(5′‐GAGAAGGCAGCCCTGGTAAC‐3′)

### 2.4 Western Blot analysis

Lungs, heart chambers and kidneys were homogenized on ice and centrifuged at 4°C for 5 minutes at 1000 *g*. The homogenized tissue was then lysed in RIPA buffer (150 mmol/L NaCl, 1% NP40, 50 mmol/L Tris pH 8.0, 0.5% sodium deoxycholate and 0.1% SDS) supplemented with a cocktail of protease inhibitors (Roche) in rotation at 4°C for 20 minutes, and then centrifuged at 4°C for 10 minutes at 13300 *g*. The cleared supernatant was collected, and protein concentration was determined (Bradford reagent, Sigma). Equal amounts of extracted proteins (40‐60 μg) were loaded and run on a 9% SDS‐polyacrylamide gel and were transferred to nitrocellulose membrane. Membranes were incubated in blocking buffer, TBS‐T (Tris‐buffered saline, 0.1% Tween 20) containing 5% (w/v) BSA, and probed with the appropriate primary antibodies: anti‐ACE2 (1:1000, goat, AF933, R&D Systems), anti‐Furin (1:1000, rabbit, ab183495, Abcam), anti‐TMPRSS2 (1:500, rabbit, ab92323, Abcam), anti‐ADAM17 (1:1000, rabbit, GTX101358, GeneTex) and anti‐GAPDH (1:500, mouse, sc‐32233, Santa Cruz). After washing with TBS‐T, the immunoreactive proteins were visualized with horseradish‐conjugated goat anti‐rabbit (1:25 000, 111‐035‐144, Jackson), donkey anti‐mouse (1:10 000, 715‐035‐151, Jackson) and donkey anti‐goat (1:10 000, 705‐035‐003, Jackson) IgG secondary antibodies and chemiluminescent substrate.

### 2.5 Tissue fixation and immunofluorescence

Additional groups of rats with compensated and decompensated CHF as well as sham controls (n = 3‐6) were anesthetized and their hearts, lungs and kidneys were fixed via right ventricle perfusion, first with 120 mL phosphate buffered saline (0.01 mol/L PBS, pH 7.4) containing heparin (5 U/mL), then with 220 mL of ice‐cold 4% paraformaldehyde in 0.01 mol/L PBS, pH 7.4 containing sucrose 4%. Heart, lungs and kidneys from the different experimental groups were removed and embedded in 0.01 mol/L PBS, pH 7.4 containing sucrose 4% and paraformaldehyde 4%. The tissues were then progressively dehydrated in graduated alcohol concentrations (70%‐100%) and embedded in paraffin. Five micrometre‐thick paraffin sections of the various tissues were deparaffinized and rehydrated. Then slides were subjected to antigen retrieval by using Proteinase K (ab64220, Abcam) for 5 minutes. Slides were then incubated with 5% normal donkey serum (NDS) in phosphate buffered saline (PBS) containing 0.3% Tween‐20 for 60 minutes to block nonspecific binding and incubated overnight at 4°C with primary antibodies diluted in blocking solution and directed against ACE2 (AF933, R&D systems), Furin (ab183495, Abcam) and TMPRSS2 (sc‐515727, Santa Cruz). Cy™3 Donkey Anti‐Rabbit IgG, Cy™3 Donkey Anti‐Goat IgG and Cy™3 Donkey Anti‐Mouse IgG were used as secondary antibodies (Jackson Laboratories) together with DAPI Fluoromount‐G^®^ for nuclear staining. Images were captured using a Widefield Zeiss Upright microscope and analysed with Zen software. Representative images of the cardiac, pulmonary and renal tissues were obtained at 40×, 20× and 20× magnification, respectively.

### 2.6 Plasma levels of ACE2 and furin

Plasma levels of ACE2 and furin of the various experimental groups were determined by commercial ELISA kits from Aviva Systems Biology, Corp.

### 2.7 Serum creatinine and blood urea nitrogen

Serum creatinine (SCr) and blood urea nitrogen (BUN) in serum samples of the various experimental groups were determined using commercial kits (Siemens) with an auto‐analyser dedicated instrument (Dimension RXL, Siemens).

### 2.8 Statistical analysis

Data are presented as mean ± SEM One‐way analysis of variance (ANOVA) for repeated measures, followed by Tukey's test for comparison of corresponding values between the groups. Comparisons between two groups were done using Student's *t* test. A value of *P* < .05 was considered statistically significant.

## 3 RESULTS

In line with the distinct UNaV pattern, SCr levels were significantly higher in compensated (1.00 ± 0.026 mg/dL, *P* < .05) and decompensated (1.04 ± 0.032 mg/dL, *P* < .01) animals than in sham controls (0.88 ± 0.03 mg/dL). Similarly, BUN levels were enhanced in compensated (25.03 ± 7.44 mg/dL, *P* < .05) and decompensated (38.53 ± 6.71 mg/dL; *P* < .01) animals as compared with sham controls (11.04 ± 0.53 mg/dL). These results are in line with our previous report^37^ that rats with CHF exhibited impaired kidney function and attenuated renal hemodynamic as compared with sham controls. Specifically, rats with decompensated CHF have significantly lower glomerular filtration rate (GFR) (0.89 ± 0.12 mL/min, *P* < .01) and renal plasma flow (RPF) (2.17 ± 0.19 mL/min, *P* < .01) as compared with compensated animals (1.57 ± 0.21 and 2. 81 ± 0.14 mL/min) and sham controls (1.96 ± 0.16 and 6.02 ± 0.29 mL/min).

**Figure 1:**
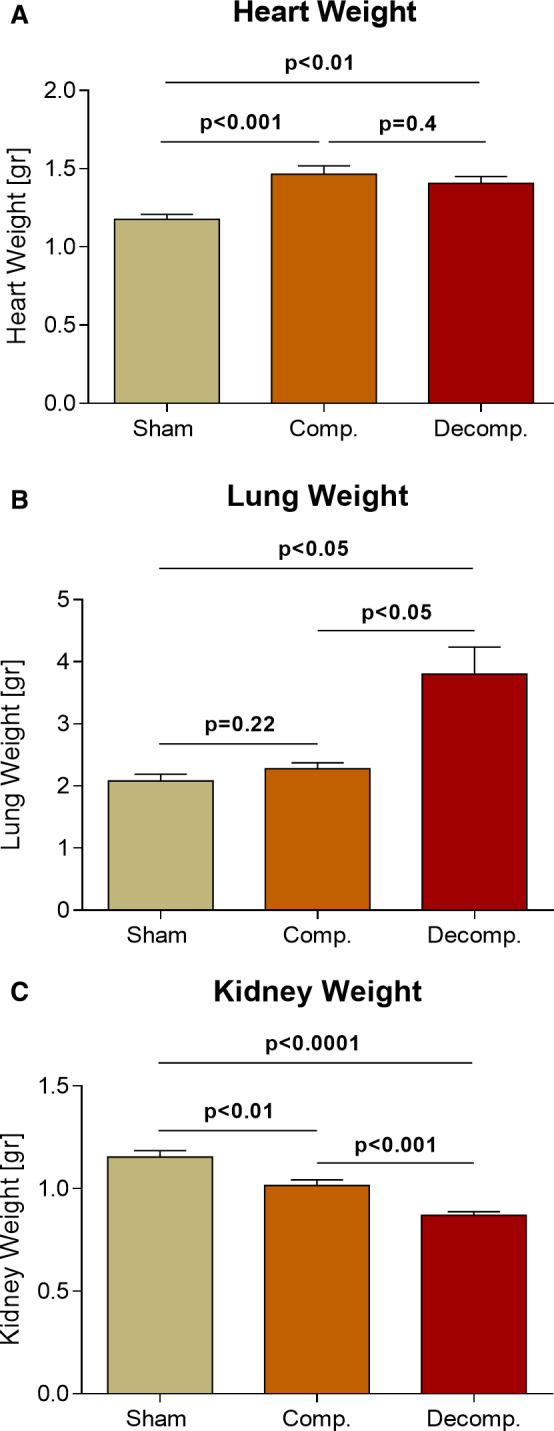
Impact of aorto‐caval placement on heart, lung and kidney weights as compared with sham‐operated controls. Cardiac, lung, and kidney weights expressed as absolute values (A–C, respectively) of rats with compensated and decompensated CHF and their sham controls (n = 9‐11). Values are means ± SEM

Rats with compensated and decompensated CHF displayed a significant increase in heart weight in comparison with their sham controls (1.465 ± 0.05 g; *P* < .001, 1.405 ± 0.044 g; *P* < .01 vs 1.175 ± 0.033 g, respectively) (Figure 1A). In addition, rats with decompensated CHF exhibited pulmonary congestion as was evident by a significant increase in lung weight as compared with sham controls (3.80 ± 0.43 g; *P* < .05 vs 2.08 ± 0.11 g, respectively) and even compensated CHF subgroup (2.28 ± 0.097 g, *P* < .05) (Figure 1B). In contrast with cardiac hypertrophy and pulmonary congestion, kidney weight was significantly declined in correlation with CHF severity, reaching 1.016 ± 0.03 g (*P* < .01) in compensated CHF and 0.871 ± 0.02 g (*P* < .0001) in decompensated CHF animals, as compared to sham controls (1.154 ± 0.03 g) (Figure 1C).

Expression and abundance of ACE2, Furin, TMPRSS2 and ADAM17 were determined in pulmonary, cardiac and renal tissues of compensated and decompensated CHF rats and sham control animals by using western blot, RT‐PCR and immunohistochemical techniques.

### 3.1 Angiotensin converting enzyme‐2

**Figure 2:**
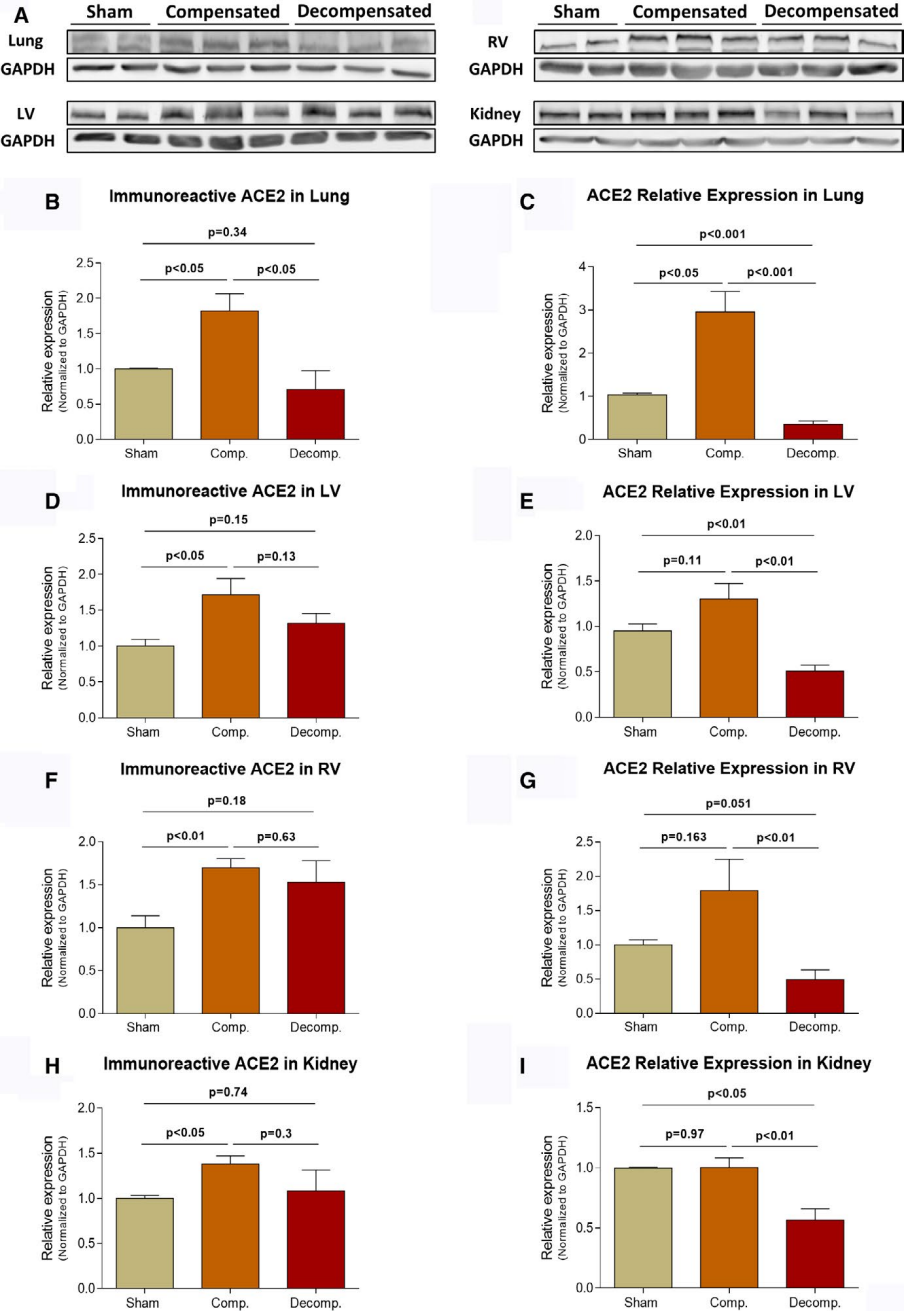
Immunoreactive levels and expression of ACE2 in the lungs, LV, RV and kidneys of compensated, decompensated CHF and sham controls as were determined with western blots analysis and RT‐qPCR. A, Representative western‐blot analysis of tissue lysates with antibody for ACE2. Western‐blot analysis quantification of ACE2 in the lungs, LV, RV and kidneys (B, D, F and H, respectively) of sham, compensated CHF and decompensated CHF (n = 3‐10), where GAPDH was used as loading control. Quantification of RT‐qPCR analysis for ACE2 mRNA normalized to GAPDH are depicted in C, E, G and I. Values are means ± SEM

Higher immunoreactive levels of ACE2 were detected in the lungs of compensated (1.82 ± 0.245, *P* < .05), but not decompensated rats (0.71 ± 0.267), as compared to sham controls (1.0 ± 0.01) (Figure 2B). In line with these findings, pulmonary ACE2 expression was ~3‐fold higher in compensated rats (2.96 ± 0.47, *P* < .05) as compared with control animals (1.03 ± 0.04) (Figure 2C). In contrast, decompensated subgroup displayed significantly lower levels of ACE2 mRNA (0.35 ± 0.08, *P* < .001) than both compensated and sham control animals (Figure 2C).

Like pulmonary tissue, ACE2 immunoreactivity in the left ventricle (LV) was enhanced in compensated and decompensated CHF subgroups as compared to sham controls (1.72 ± 0.23; *P* < .05, 1.32 ± 0.14; *P* = NS vs 1.0 ± 0.01, respectively) (Figure 2D). Similarly, ACE2 abundance in the right ventricle was enhanced in compensated, but not decompensated CHF animals (1.7 ± 0.11; *P* < .01, 1.53 ± 0.25; *P* = NS vs 1.0 ± 0.14, respectively) (Figure 2F). Interestingly, the expression of ACE2 in the LV and RV was upregulated in compensated CHF but downregulated in decompensated animals (Figure 2E,G). No differences were found in atrial immunoreactive levels of ACE2 between sham controls, compensated and decompensated CHF, although ACE2 mRNA was significantly higher in compensated in both atria as compared with decompensated subgroup and sham controls (Data not shown).

Renal ACE2 immunoreactivity was significantly higher in compensated CHF animals as compared to sham (1.38 ± 0.09 vs 1.0 ± 0.03; *P* < .05, respectively) (Figure 2H). However, the expression of ACE2 in the kidney of decompensated animals (0.57 ± 0.09) was significantly lower than that of sham controls (1.0 ± 0.08; *P* < .05) and compensated subgroup (1.0 ± 0.08; *P* < .01) (Figure 2I). Interestingly, analysis of ACE2 immunoreactivity in the renal cortex and medulla revealed again upregulation in the renal cortical tissue (1.66 ± 0.14 vs 1.0 ± 0.03; *P* < .01) as well as in the medulla (2.2 ± 0.59 vs 1.0 ± 0.11; P = NS) of compensated CHF as compared to sham controls, but not the decompensated animals (Figure S1C,D).

Immunofluorescence staining revealed intense immunostaining of ACE2 in the pulmonary, cardiac and renal tissues (Figure 6A). Specifically, ACE2 immunofluorescence was detected mainly in the endothelium of pulmonary small blood vessels and alveolar epithelial cells of all studied subgroups. In agreement with WB analysis, immunofluorescence of pulmonary ACE2 was explicitly enhanced in lung tissue of compensated CHF group, but not in decompensated CHF animals (Figure 6A). Similar pattern was observed in the cardiac tissue, where ACE2 immunofluorescence was enhanced in compensated CHF and to a lesser extent in the decompensated subgroup. Similarly, renal immunofluorescence of ACE2 was more abundant in compensated subgroup but not decompensated animals (Figure 6A). It should be emphasized that ACE2 was mainly localized to the peritubular capillaries and almost absent in tubular epithelial cells. In the next step, we analysed regional ACE2 immunofluorescence in the kidney. ACE2 immunofluorescence was localized mainly to the renal cortex (vasculature) and to lesser extent in the medulla (vasa recta) (Figure S2A).

### 3.2 Furin

**Figure 3:**
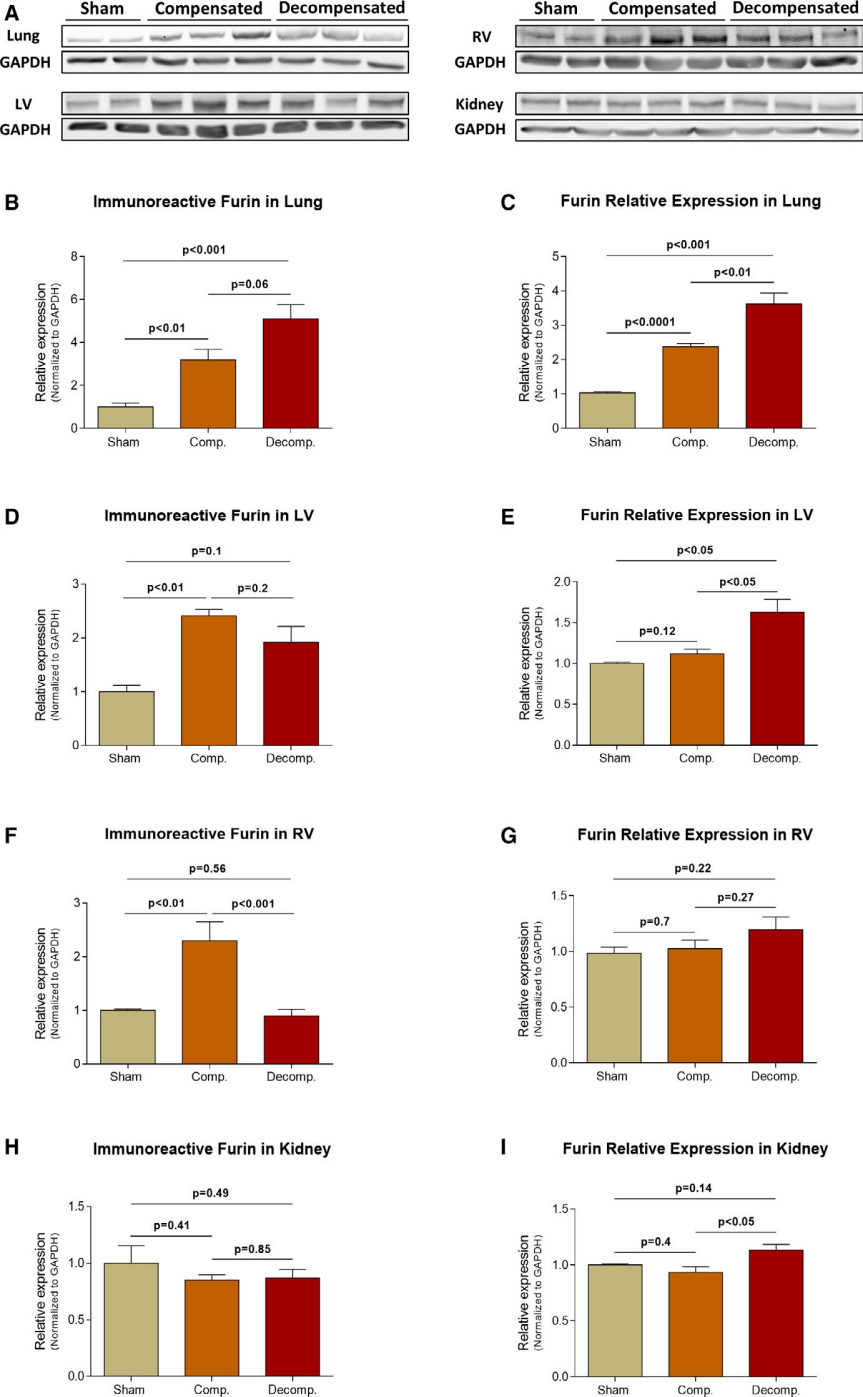
Immunoreactive levels and expression of furin in the lungs, LV, RV and kidneys of compensated, decompensated CHF and sham controls as were determined with western blots analysis and RT‐qPCR. A, Representative western blot analysis of tissue lysates with antibody for furin. Western blot analysis quantification of furin in the lungs, LV, RV and kidneys (B, D, F and H, respectively) of sham, compensated CHF and decompensated CHF (n = 3‐11), where GAPDH was used as loading control. Quantification of RT‐qPCR analysis for furin mRNA normalized to GAPDH are depicted in C, E, G and I. Values are means ± SEM

Figure 3 depicts the immunoreactive levels and expression of furin in the pulmonary, cardiac and renal tissues. Furin was detected in significantly higher levels in lung homogenates of both compensated CHF rats and decompensated CHF animals compared with sham controls (3.19 ± 0.5; *P* < .01, 5.1 ± 0.66; *P* < .001 vs 1.0 ± 0.19, respectively) (Figure 3B). In line with these findings, pulmonary furin expression was ~2.5 and ~3.5 fold higher in compensated and decompensated rats as compared with control group, respectively (2.38 ± 0.1; *P* < .0001, 3.62 ± 0.32; *P* < .001 vs 1.03 ± 0.03, respectively) (Figure 3C).

Like pulmonary tissue, furin immunoreactivity in the LV was significantly enhanced in compensated and to a lesser extent in decompensated CHF subgroup as compared to sham controls. Specifically, furin immunoreactive levels in LV were 2.41 ± 0.12 fold (*P* < .01) and 1.92 ± 0.3 fold (*P* = NS) in compensated and decompensated subgroups, respectively (Figure 3D). Similar trend was detected in RV of compensated (2.3 ± 0.35; *P* < .01), but not of decompensated (0.895 ± 0.12; *P* = NS) CHF subgroup, when compared to sham‐operated rats (1.0 ± 0.03) (Figure 3F).

Renal furin immunoreactive levels and expression are presented in Figure 3H,I. Neither the abundance nor the expression of furin in the kidney have changed following the induction of CHF. Analysis of regional furin immunoreactivity in kidney unravelled slight upregulation of cortical furin (1.36 ± 0.12; *P* < .05), but not in the medulla (0.93 ± 0.12; *P* = NS) of compensated CHF as compared to sham controls (1.0 ± 0.01) (Figure S1E,F).

Immunofluorescence staining revealed intense immunostaining of furin in the pulmonary, cardiac and renal tissues (Figure 6B). Specifically, furin immunofluorescence was detected in the alveolar epithelial cells of all studied groups. Noteworthy, immunostaining was more intense in rats with compensated and decompensated CHF (Figure 6B). In agreement with the WB and RT‐PCR analysis, immunofluorescence of furin was enhanced in cardiac tissue of both compensated and decompensated CHF subgroups (Figure 6B). Renal immunofluorescence of furin was abundant in the tubular epithelial cells and vascular endothelium, where it is elevated in the compensated group and to a lesser extent in the decompensated group, compared to sham rats. (Figure 6B). Analysis of regional furin immunofluorescence showed abundance of this enzyme in both renal cortex and medulla, mainly in the tubule. Induction of CHF slightly enhanced furin immunofluorescence in the cortical and medullary tissues (Figure S2B).

### 3.3 TMPRSS2

**Figure 4:**
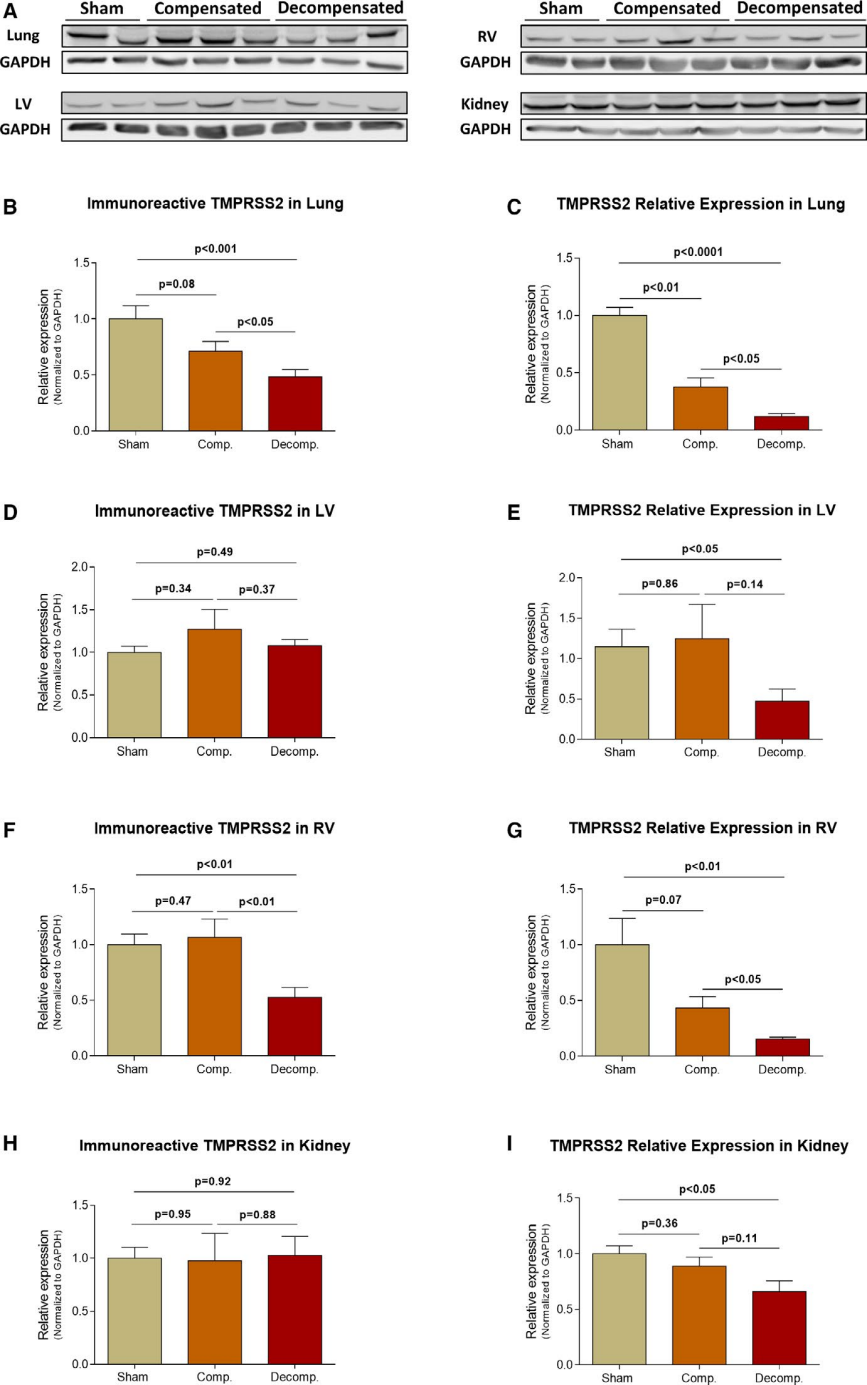
Immunoreactive levels and expression of TMPRSS2 in the lungs, LV, RV and kidneys of compensated, decompensated CHF and sham controls as were determined with western blots analysis and RT‐qPCR. A, Representative western‐blot analysis of tissue lysates with antibody for TMPRSS2. Western‐blot analysis quantification of TMPRSS2 in the lungs, LV, RV and kidneys (B, D, F and H, respectively) of sham, compensated CHF and decompensated CHF (n = 3‐10), where GAPDH was used as loading control. Quantification of RT‐qPCR analysis for TMPRSS2 mRNA normalized to GAPDH are depicted in C, E, G and I. Values are means ± SEM

The immunoreactive levels and expression of TMPRSS2 in the pulmonary, cardiac and renal tissues are presented in Figure 4. The immunoreactive levels of TMPRSS2 declined in the pulmonary tissue of CHF animals in correlation with the severity of the disease, where it reached 48.3 ± 6% (*P* < .001) in decompensated subgroup (Figure 4B). In parallel to these results, pulmonary TMPRSS2 mRNA levels were significantly decreased in both compensated and decompensated CHF animals (0.38 ± 0.08; *P* < .01, 0.12 ± 0.03; *P* < .0001 vs 1.0 ± 0.07, respectively), where the decline was more profound in the latter subgroup (Figure 4C). While LV immunoreactive levels of TMPRSS2 was not significantly changed following CHF induction (Figure 4D), RV TMPRSS2 abundance was decreased in decompensated CHF animals by ~ 53±9% (*P* < .01) (Figure 4F). In RV, and in similarity with its behaviour in the lung and LV tissues, TMPRSS2 expression was downregulated in CHF animals, especially in the decompensated subgroup, in comparison with sham‐operated animals (0.15 ± 0.02 vs 1.0 ± 0.23; *P* < .01, respectively) (Figure 4G). Renal TMPRSS2 immunoreactive levels and expression are presented in Figure 4H,I. While the abundance of TMPRSS2 was not affected by CHF, the expression of this enzyme in the renal tissue declined in correlation with CHF severity, reaching 66 ± 10% (*P* < .05) in the decompensated subgroup (Figure 4I). While no changes in TMPRSS2 in the renal cortex and medulla of compensated rats as compared with sham controls were observed, decompensated animals exhibited down regulation of this enzyme in both the cortex and medulla (0.76 ± 0.08; *P* < .05 vs 1.0 ± 0.04, 0.82 ± 0.06; *P* < .05 vs 1.0 ± 0.06, respectively) (Figure S1G,H).

Immunofluorescence staining revealed immunostaining of TMPRSS2 in the pulmonary, cardiac and renal tissues (Figure 6C). Specifically, TMPRSS2 immunofluorescence was detected in the alveolar epithelial cells of sham controls and CHF subgroups (Figure 6C). Noteworthy, decompensated animals exhibited remarkable decrease in TMPRSS2 in the lung. While immunofluorescence TMPRSS2 was enhanced in cardiac tissue of compensated and to a lesser extent in decompensated CHF subgroup, in the renal tissue it was upregulated mainly in the compensated subgroup (Figure 6C). Analysis of regional TMPRSS2 immunofluorescence showed abundance of this enzyme in the renal cortex, but not in the medulla (Figure S2C). Decompensated CHF animals displayed down regulation of cortical TMPRSS2 immunofluorescence, whereas compensated CHF did not show remarkable changes.

### 3.4 ADAM17

**Figure 5:**
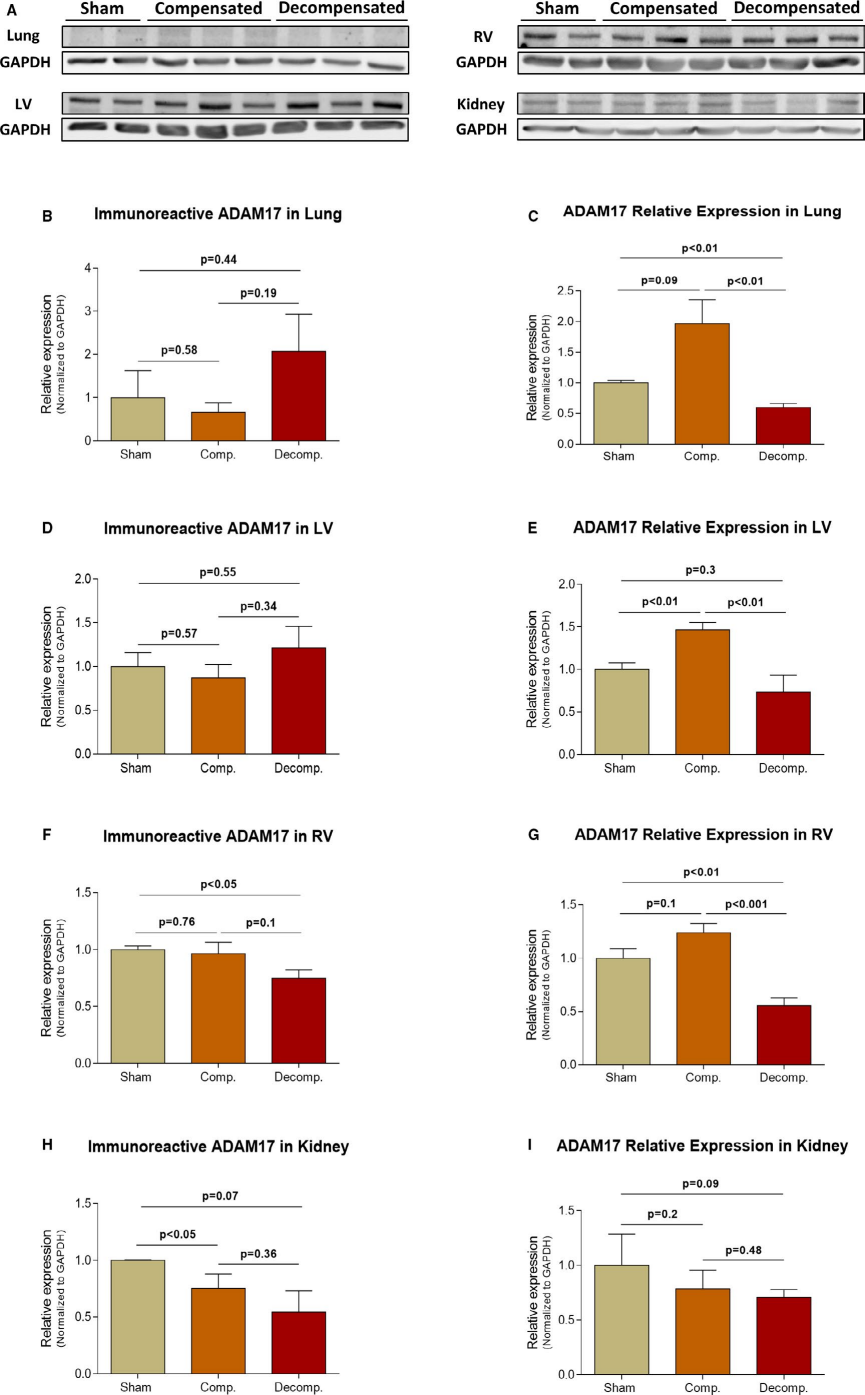
Immunoreactive levels and expression of ADAM17 in the lungs, LV, RV and kidneys of compensated, decompensated CHF and sham controls as were determined with western blots analysis and RT‐qPCR. A, Representative western blot analysis of tissue lysates with antibody for ADAM17. Western‐blot analysis quantification of ADAM17 in the lungs, LV, RV and kidneys (B, D, F and H, respectively) of sham, compensated CHF and decompensated CHF (n = 3‐10), where GAPDH was used as loading control. Quantification of RT‐qPCR analysis for ADAM17 mRNA normalized to GAPDH are depicted in C, E, G and I. Values are means ± SEM

The immunoreactive levels and expression of ADAM17 in the pulmonary, cardiac and renal tissues are presented in Figure 5. While compensated animals displayed no differences in pulmonary immunoreactive levels of ADAM17 as compared with sham controls, a non‐significant upregulation was observed in decompensated rats (Figure 5B). In contrast, pulmonary ADAM17 expression exhibited distinct pattern, where this enzyme was increased by ~2 fold (1.97 ± 0.39; *P* = .09) in compensated rats and declined by ~40% (0.6 ± 0.07; *P* < .01) in decompensated CHF subgroup (Figure 5C).

**Figure 6:**
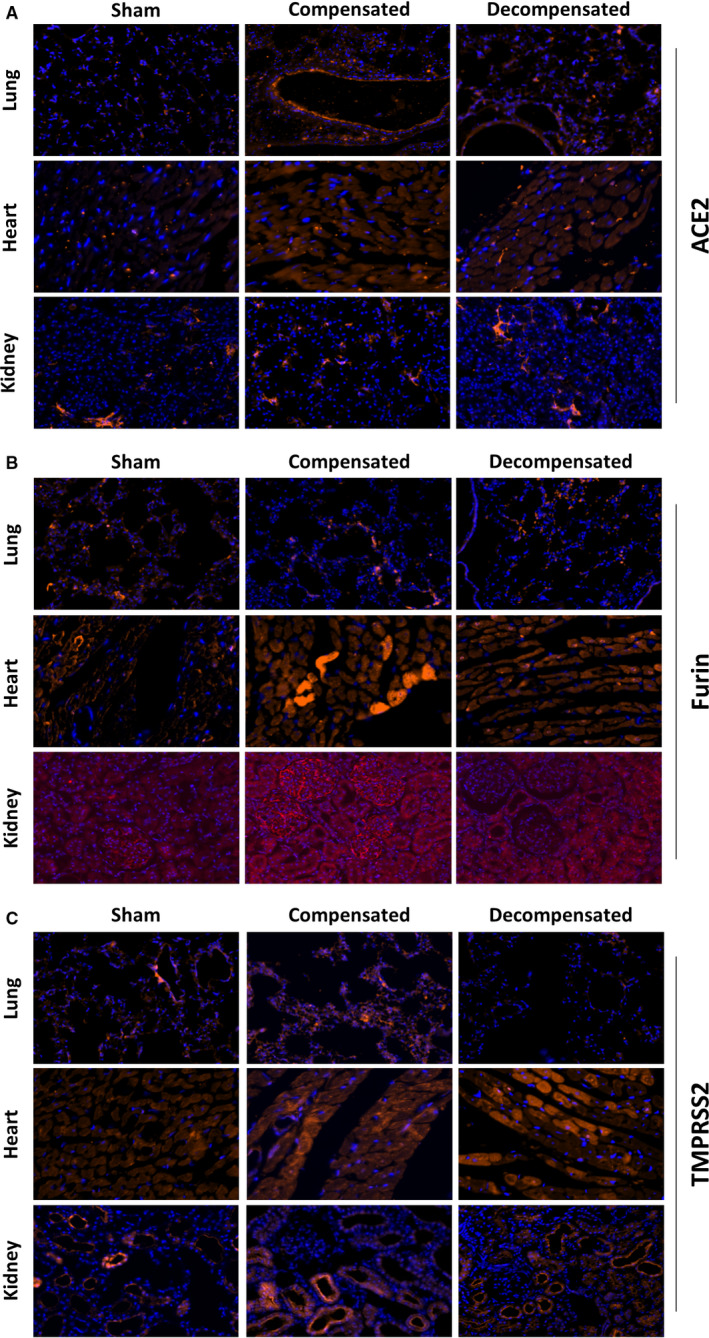
Histological changes in lung, heart and kidney tissues from rats with compensated and decompensated CHF as compared to their sham controls. Slices from lung, heart and kidneys of the various experimental groups were stained with primary antibodies of ACE2 (A), Furin (B) and TMPRSS2 (C). Cy™3 Donkey Anti‐Rabbit IgG, Cy™3 Donkey Anti‐Goat IgG and Cy™3 Donkey Anti‐Mouse IgG were used as secondary antibodies together with DAPI Fluoromount‐G^®^ for nuclear staining. Images were captured using a Widefield Zeiss Upright microscope and analysed with Zen software. Representative images of the cardiac, pulmonary and renal tissues were obtained at 40×, 20× and 20× magnification, respectively

While no change in ADAM17 immunoreactivity was detected in LV tissue of the different CHF subgroups, lower levels were observed in the RV chamber of decompensated CHF animals (0.75 ± 0.07; *P* < .05) (Figure 5D,F). The expression of ADAM17 in LV was significantly higher in compensated CHF rats as compared with sham (1.47 ± 0.08 vs 1.0 ± 0.08; *P* < .01), but lower in decompensated animals (0.74 ± 0.2 vs 1.0 ± 0.08; *P* = NS), (Figure 5E). Similarly, the expression of ADAM‐17 in RV was higher in compensated CHF rats as compared with sham (1.24 ± 0.09 vs 1.0 ± 0.09; *P* = NS), but significantly lower in decompensated animals (0.56 ± 0.07 vs 1.0 ± 0.09; *P* < .01) (Figure 5G).

Renal ADAM17 immunoreactivity declined in compensated CHF and decompensated groups in correlation with CHF severity (0.75 ± 0.07; *P* < .05, 0.54 ± 0.19; *P* = .07, vs 1.0 ± 0.01, respectively) (Figure 5H,I).

### 3.5 Plasma levels of ACE2 and furin

**Figure 7:**
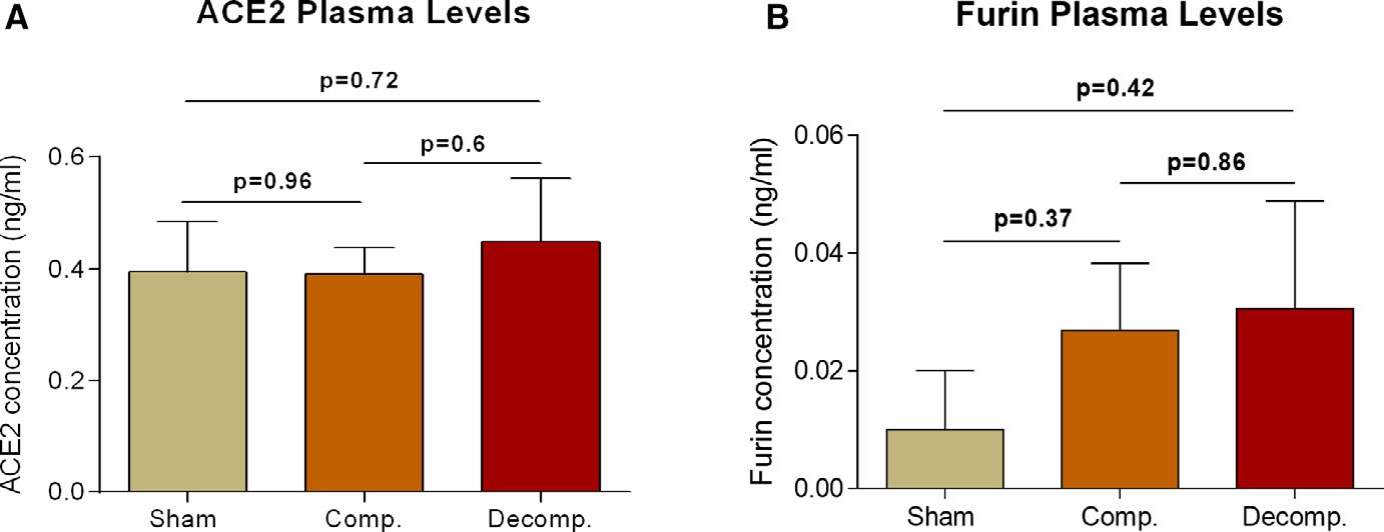
Circulatory levels of soluble ACE2 (sACE2) and furin in rats with compensated and decompensated CHF and their sham controls. Plasma samples from the various experimental groups (n = 4‐9) were collected and analysed for ACE2 (A) and furin (B) levels by utilizing commercial ELISA assays. Values are means ± SEM

Plasma levels of ACE2 and furin of the various experimental groups are shown in Figure 7. No change in ACE2 levels was detected in CHF compensated (0.39 ± 0.05 ng/mL) and decompensated (0.45 ± 0.11 ng/mL) subgroups as compared with their sham controls (0.39 ± 0.09 ng/mL). In contrast, circulatory levels of furin increased in CHF animals in correlation with the disease severity: 0.03 ± 0.01 ng/mL (Compensated) and 0.03 ± 0.02 (Decompensated) vs 0.01 ± 0.01 (Sham), but did not reach statistical significance.

## 4 DISCUSSION

Heart failure is characterized by enhanced cardiac expression of ACE2, a homing target for SARS‐CoV‐2. Since this disease state predisposes to severe COVID‐19 disease, the current study examined the impact of experimental heart failure on the expression of several key enzymes involved in SARS‐CoV‐2 attachment to target host‐cells and activation. Specifically, we determined the abundance and expression of ACE2, TMPRSS2, furin and ADAM17 in three vital organs vulnerable to this highly infectious virus, the lung, heart and kidney. Our data show that ACE2 transcription and abundance were significantly enhanced in pulmonary, cardiac and renal tissues of compensated, but not in decompensated CHF animals. Furin immunoreactivity was increased in both compensated and decompensated CHF in pulmonary and cardiac tissues, but not in the kidney. In contrast, both the expression and abundance of pulmonary, cardiac and renal TMPRSS2 decreased in CHF in correlation with the severity of the disease. Similarly, pulmonary, cardiac and renal ADAM17 mRNA levels were downregulated in rats with decompensated CHF. The increased expression of tissue‐protective ACE2 in heart failure may serve as a compensatory response to the over‐activity of the deleterious isoform, namely, ACE. Furthermore, deteriorating features of decompensated CHF might be explained by failure to reach high expression of the tissue‐protective and diuretic ACE2.

Our findings showing enhanced ACE2 in experimental compensated CHF is in line with previous studies showing its enhanced myocardial expression in patients with CHF^8^, ^16^, ^20^, ^25^ that may represent a cardioprotective compensatory response aimed at reducing or preventing cardiac remodelling.^17^ In line with this assumption, targeted overexpression of cardiac ACE2 in rats, by applying local injection of lentiviral vector, significantly attenuated cardiac hypertrophy and myocardial fibrosis induced by prolonged Ang II administration.^38^ Similarly, overexpression of ACE2 in cardiac tissues of spontaneously hypertensive rats decreased cardiac remodelling as was evident by reduced left ventricular wall thickness and perivascular fibrosis,^39^ probably via reduction of collagen synthesis.^40^ Plausible ACE2/Ang 1‐7/Mas‐R ‐mediated protective mechanisms might involve myocardial conservation, afterload‐reduction, natriuresis and diuresis.^8^, ^16^, ^20^, ^41^, ^42^ In these perspectives, our current findings, showing that enhanced myocardial ACE2 expression in compensated CHF is blunted and even suppressed in rats with decompensated CHF, suggest a role for ACE2 in maintaining viability in the setup of cardiac dysfunction. Support for this concept emerges from previous experimental reports demonstrating cardiac contractility defects in rats with reduced X chromosomal‐derived ACE2 expression and heart failure with pulmonary congestion in ACE2 knockout mice (ACE2 KO).^43^, ^44^ Interestingly, the hearts of animals depleted of ACE2 exhibited similar changes that occur after coronary artery disease or bypass surgery in humans.^45^ Subsequent studies demonstrated extended infarct size, reduced contractility, altered ventricular remodelling and increased mortality following myocardial infarction (MI) induced by ligation of the proximal LAD artery in mice with ACE2 deletion, as compared with their wildtype controls.^46^ Moreover, these mice showed enhanced oxidative stress and concomitant upregulation of proinflammatory cytokines, plausibly parallel to the observed hypersensitive immunological response reported in patients with SARS‐CoV‐2 infection.

The deleterious impact of excess Ang II over Ang 1‐7 can also be appreciated by experimental Ang II enhancement. We have previously demonstrated that RAAS including the classic ACE are activated in rats with acute cardiac failure in correlation with disease severity.^37^, ^47^ The deleterious role of the RAAS in the progression of cardiovascular, pulmonary and renal dysfunction in CHF is well established.^48^ For instance, it was shown that prolonged activation of the RAAS has direct adverse actions on the myocardium, independent of its systemic hemodynamic effects.^49^ Specifically, Ang II exerts hypertrophic effects on the myocytes and enhances fibrosis and apoptosis, resulting eventually into progressive remodelling and further deterioration in cardiac performance.^50^ Besides the myocardium, the RAAS is profoundly involved in pulmonary oedema formation^51^ and kidney dysfunction,^37^ hallmark features of CHF.

Our findings may bear relevance to the particular susceptibility of patients with CHF to SARS‐CoV‐2 infection. Overexpression of ACE2 and furin in the pulmonary, cardiac and renal tissues of compensated as compared with their sham controls, suggests their potential role in viral attachment and virulence, predominantly in this setup. By contrast, the substantial downregulation of TMPRSS2 and ADAM17 in experimental CHF argues against the involvement of these enzymes in the exaggerated susceptibility of CHF patients to attract SARS‐CoV‐2.

Furthermore, it has been suggested that COVID‐19 disease severity reflects imbalanced effects of Ang II/AT‐1 and Ang 1‐7/Mas‐R at tissue level, promoting vasoconstriction, inflammation and coagulopathy. It is therefore tempting to assume that the enhanced vulnerability of severe, decompensated CHF to COVID‐19 manifestations may stem from an inadequate ACE2 compensatory response along with a depletion of the tissue‐protective Ang 1‐7, leading to an unrestricted stimulation of the harmful arm of RAAS, that is, ACE/Ang II/AT‐1. Collectively, the present study provides new insights regarding plausible pathophysiologic mechanisms and susceptibility to COVID‐19 infection in general, and specifically in the setup of CHF.

As was already shown for SARS‐CoV‐1, SARS‐CoV‐2 viral spike glycoprotein binds with high affinity to ACE2 where it triggers its internalization along with the virus.^28^, ^52^, ^53^, ^54^, ^55^ The deleterious effects of SARS‐CoV‐2 are largely attributed to the deprivation of the affected target cells from their advantageous ACE2‐Ang 1‐7‐MasR machinery. Once attachment of SARS‐CoV‐2 to its target cell is achieved by binding to ACE2, facilitated by furin, a vicious feed‐forward cycle develops with TMPRSS2 facilitating internalization of the virus‐ACE2 complex and stimulation of ADAM17‐mediated detachment of ACE2 from the cell membrane into the blood stream (as detailed below), collectively leading to membranal ACE2 depletion and to the suppression of Ang 1‐7 syntheses, leaving the deleterious impact of Ang II unopposed.^6^ This ultimately leads to SARS‐CoV‐2‐induced progressive lung injury with interstitial and non‐cardiogenic pulmonary oedema, inflammation, necrosis, fibrosis and disseminated clotting. The same pattern of injury likely develops in other vital organs, such as the myocardium or kidneys. Indeed, corona virus has already been shown to induce myocardial inflammation and dysfunction accompanied with adverse cardiac outcomes in patients with SARS, assumedly due to downregulation/elimination of myocardial ACE2.^23^ Noteworthy, SARS‐CoV‐2 and its traces were detected in the myocardium and even the renal tissue, suggesting potential involvement of SARS‐CoV‐2 in renal and cardiac manifestations of COVID‐19,^56^, ^57^, ^58^, ^59^ although this matter requires additional research. Therefore, it is tempting to assume that an appropriate upregulation of cardiac, pulmonary and renal ACE2, along enhanced Ang 1‐7 production may represent an advantageous compensatory response in compensated CHF facing SARS‐CoV‐2 infection, whereas failure of ACE2 upregulation in decompensated CHF may contribute to the pathogenesis of heart failure and aggravates their COVID‐19 manifestations due to the lack of adequate Ang 1‐7 production. In this context, a recent study by Shovlin et al^60^ demonstrated that failure to suppress replication of the SARS‐CoV‐2 virus within five days, results in sustained downregulation of ACE2. Hampered ACE2 upregulation predicts subsequent catastrophic outcomes, including impaired pulmonary endothelial homeostasis. These findings suggest that downregulation of ACE2 by itself plays a harmful role during SARS‐CoV‐2 infection, independently of the fact that ACE2 serves as receptor of this virus.

As mentioned above, ADAM17 is another important player in the control of membranal density of ACE2. Activated by Ang II, ADAM17 increases the shedding of ACE2 to the circulation (sheddase activity), thereby depleting membranal ACE2 and increasing its plasma levels.^36^ ADAM17 depletion in decompensated CHF might serve in promoting membranal ACE2 availability, together with enhanced ACE2 synthesis. However, plasma free ACE2 levels in rats with both compensated and non‐compensated CHF were not different from controls, suggesting that variations in ACE2 shedding from cell membranes did not significantly affect membrane‐bound ACE2 tissue levels, rather likely reflecting its transcription. Our findings regarding ADAM17 transcription varies in the different organs and between the CHF phenotypes. Pulmonary and cardiac ADAM17 mRNA levels were upregulated in compensated CHF but downregulated in decompensated subgroup. In contrast, in the kidney ADAM17 expression/abundance was decreased in correlation with the heart failure severity. The downregulation of renal ADAM17 in rats with CHF, may explain the relatively limited incidence of acute kidney injury (AKI) in COVID‐19 disease, as opposed to the substantial pulmonary and myocardial injuries. Perhaps, declining ADAM17 renal expression in CHF preserves abundance of ACE2 in renal tissues despite its internalization and degradation, promoting Ang 1‐7 excess over Ang II in pericytes, as the upregulation of ACE2 in the pulmonary, cardiac and renal tissues may contribute to maintain the integrity of these vital systems in COVID‐19 disease.^17^, ^42^, ^61^ In addition, the unopposed activity of Ang II (by Ang 1‐7) may have the potential for deleterious effects, in patients suffering from pneumonitis or background diseases such as heart failure^6^ or diabetes.^5^, ^41^ It has been demonstrated that in acute lung injury ACE2 is downregulated, therefore leading to unopposed Ang II that may enhance lung injury.^62^

Heart failure is also associated with increased expression of furin.^28^ Furin presents mainly intracellularly and to a lesser extent in the circulation,^33^ where it converts ventricular proBNP to active BNP, an important physiological process in heart failure subjects. Patients with heart failure are specifically characterized by upregulation of cardiac furin, providing an additional potential explanation for their vulnerability COVID‐19 infection.^63^ Furthermore, CHF animals displayed high levels of circulatory furin, in correlation with the CHF severity. This may form a feed‐forward loop of furin‐facilitated coronavirus replication, leading to fulminant myocarditis, devastating lung injury and lethal multi‐organ failure. In line with this assumption, rats with CHF displayed upregulation of furin in the heart and lung, but not the kidney. The upregulation was obtained in both compensated and decompensated CHF subgroups. However, it could not be excluded that the observed increase in furin during CHF may represent a sole compensatory response to meet the additional need for BNP required to counterbalance the fibrotic and sodium retaining deleterious actions of the exaggerated levels of Ang II and aldosterone, characterizing this clinical setting.

Our study is descriptive and does not address mechanisms involved in the changes in the transcription and expression of ACE2 and of the molecules linked to ACE2 regulation caused by CHF. Further studies are needed to assess their status in humans with CHF. Furthermore, there is no data regarding their expression and role in humans during COVID‐19 disease, and our relevant discussion is to large extent speculative. Yet these assumptions and suggestions should be further evaluated, since they may bear clinical relevance with plausible interventional implications in this devastating disease. Additional limitation of the current study is the short follow up period (1 week), which represents acute CHF, rather than a chronic condition that usually develops in humans in many years.

**Figure 8:**
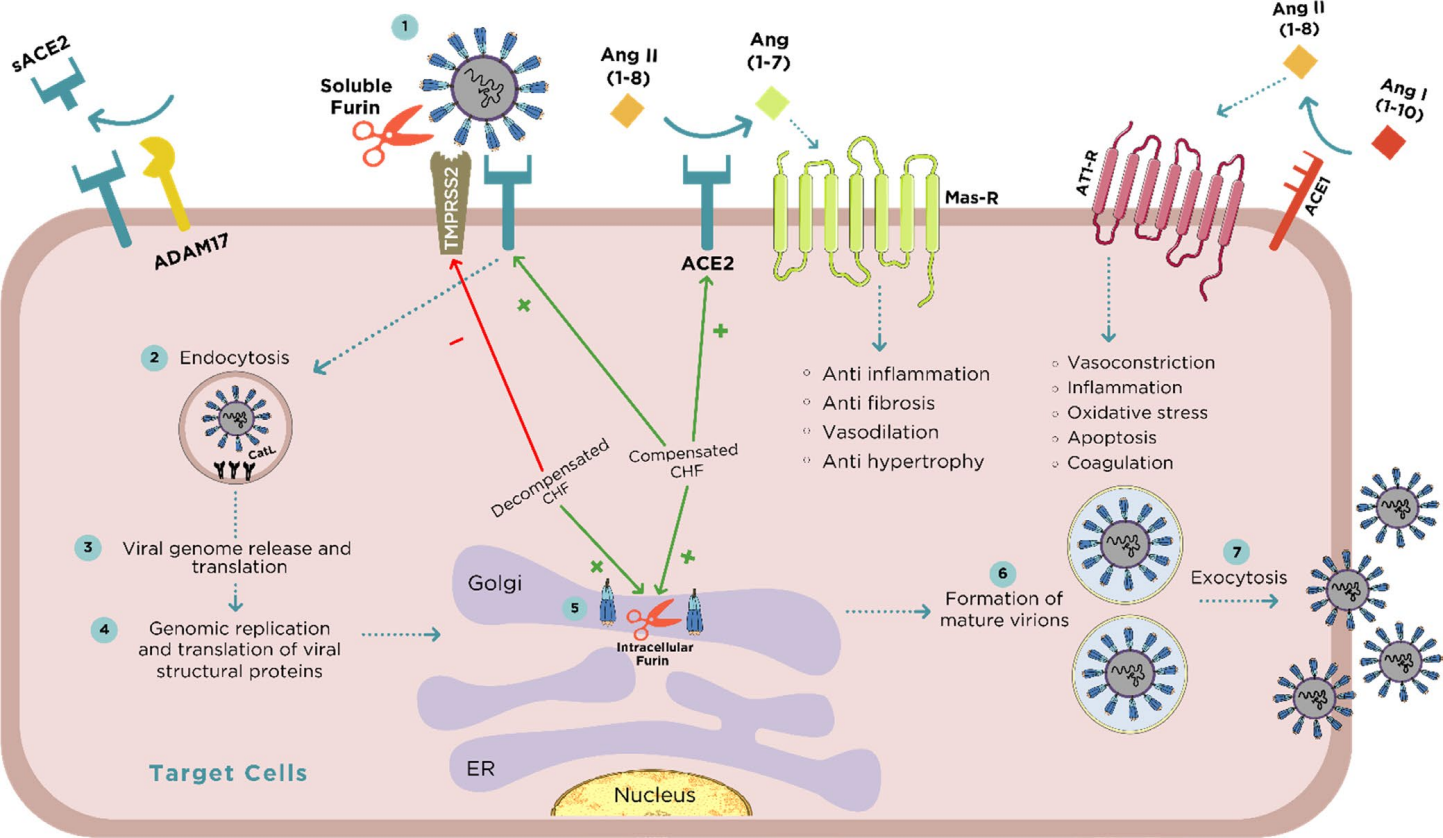
SARS‐CoV‐2 binding, activation, invasion and replication in target cells. The initial step after the invasion of SARS‐CoV‐2 is binding to membranal ACE2 widely expressed in vital organs including lung, heart and kidney. ACE2 is responsible for the conversion of Ang II to Ang 1‐7 which exerts beneficial effects on the cardiac tissue such as vasodilation, anti‐fibrosis and anti‐inflammation via Mas receptor (MasR). The binding of SARS‐CoV‐2 to ACE2 is preceded by TMPRSS2/furin‐mediated exposure of the viral receptor binding protein (RBP) localized to S‐glycoprotein (S1 domain of the viral spike) and revealing the viral effusion site on S2 domain. Furin is expressed in these tissues both intracellularly and in the circulation as a free enzyme, making it a key factor along TMPRSS2 in the uncovering of RBP and eventually in SARS‐CoV‐2 transmission. In addition, furin enhances the affinity of the virus to ACE2, not only by exposing the viral binding site on S1 domain but also by revealing the effusion site on the S2 domain in the viral spike. Consequently, the virus undergoes endocytosis and massive replication accompanied by profound activation by the abundant intracellular furin and Cathepsin L (Cat‐L). The activated intracellular SARS‐CoV‐2 undergoes exocytosis where it binds again to ACE2 elsewhere, thus creating a vicious feed‐forward devastating cycle. According to the current study, compensated, but not decompensated congestive heart failure (CHF) is characterized by enhanced expression of myocardial ACE2 and downregulation of TMPRSS2. ADAM17 is responsible for shedding of ACE2, a process stimulated by AT1 receptor (AT1‐R) and may explain why renin angiotensin aldosterone system (RAAS) inhibitors augment ACE2 expression.

In summary, ACE2 and furin are overexpressed in pulmonary, cardiac and renal tissues of compensated and to a lesser extent of decompensated CHF as compared with their sham controls (Figure 8). While upregulation of myocardial ACE2 in heart failure has been reported in animals and humans,^8^, ^16^, ^20^, ^25^ its enhanced expression in pulmonary and renal tissues, reported here, is a novel finding. The increased expression of the beneficial ACE2 and of furin in compensated heart failure may serve as a compensatory response to the over‐activity of the deleterious isoform, namely, ACE. Failure to increase ACE2 might play a role in the development of decompensated CHF, through augmented afterload, ROS‐mediated myocardial injury, cardiac remodelling and fibrosis and fluid retention (Figure 8). In the perspective of the role ACE2 plays in SARS‐CoV‐2 invasion and the likely impact of its depletion in the clinical manifestations of COVID‐19 disease, the observed additional changes in TMPRSS2 and ADAM17 tissue expression in experimental CHF may have pathophysiologic significance.

## CONFLICT OF INTEREST

Authors have no conflicting interests.

## AUTHOR CONTRIBUTIONS

**Emad E. Khoury:** Conceptualization (equal); Data curation (equal); Formal analysis (equal); Investigation (equal); Methodology (equal); Project administration (supporting); Software (equal); Validation (equal); Visualization (equal); Writing‐original draft (equal); Writing‐review & editing (equal). **Yara Knany:** Conceptualization (equal); Data curation (equal); Formal analysis (equal); Investigation (equal); Methodology (equal); Software (equal); Validation (equal); Visualization (equal); Writing‐original draft (equal). **Ahmad Fokra:** Data curation (supporting); Formal analysis (supporting); Investigation (supporting); Methodology (supporting); Software (equal); Writing‐original draft (supporting). **Safa Kinaneh:** Data curation (supporting); Formal analysis (supporting); Investigation (supporting); Methodology (lead); Software (supporting); Visualization (supporting); Writing‐original draft (supporting). **Zaher Azzam:** Conceptualization (supporting); Data curation (supporting); Formal analysis (supporting); Investigation (supporting); Methodology (supporting); Project administration (supporting); Resources (supporting); Writing‐original draft (supporting). **Samuel N. Heyman:** Conceptualization (supporting); Formal analysis (supporting); Investigation (supporting); Validation (supporting); Visualization (supporting); Writing‐review & editing (equal). **Zaid Abassi:** Conceptualization (lead); Data curation (lead); Formal analysis (equal); Funding acquisition (lead); Investigation (lead); Methodology (equal); Project administration (lead); Resources (lead); Software (supporting); Supervision (lead); Validation (equal); Visualization (equal); Writing‐original draft (lead); Writing‐review & editing (equal).

## Supporting information

Fig S1

Fig S2

## Data Availability

The data that support the findings of this study are available on request from the corresponding author. The data are not publicly available due to privacy or ethical restrictions.

## References

[1] Epidemiology Working Group for Ncip Epidemic Response CCfDC, Prevention . The epidemiological characteristics of an outbreak of 2019 novel coronavirus diseases (COVID‐19) in China. Zhonghua liu xing bing xue za zhi. 2020;41(2):145‐151. 32064853 10.3760/cma.j.issn.0254-6450.2020.02.003

[2] Turner AJ , Hiscox JA , Hooper NM . ACE2: from vasopeptidase to SARS virus receptor. Trends Pharmacol Sci. 2004;25(6):291‐294. 15165741 10.1016/j.tips.2004.04.001 PMC7119032

[3] Bonow RO , Fonarow GC , O'Gara PT , Yancy CW . Association of coronavirus disease 2019 (COVID‐19) with myocardial injury and mortality. JAMA Cardiol. 2020.5(7):751–753. 32219362 10.1001/jamacardio.2020.1105

[4] Liu PP , Blet A , Smyth D , Li H . The science underlying COVID‐19: implications for the cardiovascular system. Circulation. 2020;142(1):68‐78. 32293910 10.1161/CIRCULATIONAHA.120.047549

[5] Fang L , Karakiulakis G , Roth M . Are patients with hypertension and diabetes mellitus at increased risk for COVID‐19 infection? (vol 8, pg e21, 2020). Lancet Respir Med. 2020;8(6):E54‐E54. 32171062 10.1016/S2213-2600(20)30116-8 PMC7118626

[6] Lother SA , Abassi M , Agostinis A , et al. Post‐exposure prophylaxis or pre‐emptive therapy for severe acute respiratory syndrome coronavirus 2 (SARS‐CoV‐2): study protocol for a pragmatic randomized‐controlled trial. Can J Anaesth. 2020;67(9):1201‐1211. 32383125 10.1007/s12630-020-01684-7 PMC7205369

[7] Abassi ZA , Skorecki K , Heyman SN , Kinaneh S , Armaly Z . Reply to Letter to the Editor: "COVID‐19: is the ACE2 just a foe?". Am J Physiol Lung Cell Mol Physiol. 2020;318(5):L1031. 32364443 10.1152/ajplung.00134.2020 PMC7203571

[8] Chen L , Li XJ , Chen MQ , Feng Y , Xiong CL . The ACE2 expression in human heart indicates new potential mechanism of heart injury among patients infected with SARS‐CoV‐2. Cardiovasc Res. 2020;116(6):1097‐1100. 32227090 10.1093/cvr/cvaa078 PMC7184507

[9] Zhou P , Yang XL , Wang XG , et al. A pneumonia outbreak associated with a new coronavirus of probable bat origin. Nature. 2020;579(7798):270‐273. 32015507 10.1038/s41586-020-2012-7 PMC7095418

[10] Xu X , Chen P , Wang J , et al. Evolution of the novel coronavirus from the ongoing Wuhan outbreak and modeling of its spike protein for risk of human transmission. Sci China Life Sci. 2020;63(3):457‐460. 32009228 10.1007/s11427-020-1637-5 PMC7089049

[11] Zou X , Chen K , Zou J , Han P , Hao J , Han Z . Single‐cell RNA‐seq data analysis on the receptor ACE2 expression reveals the potential risk of different human organs vulnerable to 2019‐nCoV infection. Front Med. 2020;14(2):185‐192. 32170560 10.1007/s11684-020-0754-0 PMC7088738

[12] Zhao Y , Zhao Z , Wang Y , Zhou Y , Ma Y , Zuo W . Single‐cell RNA expression profiling of ACE2, the receptor of SARS‐CoV‐2. bioRxiv: the preprint server for biology 2020:2020.2001.2026.919985.

[13] Tipnis SR , Hooper NM , Hyde R , Karran E , Christie G , Turner AJ . A human homolog of angiotensin‐converting enzyme. Cloning and functional expression as a captopril‐insensitive carboxypeptidase. J Biol Chem. 2000;275(43):33238‐33243. 10924499 10.1074/jbc.M002615200

[14] Hamming I , Timens W , Bulthuis ML , Lely AT , Navis G , van Goor H . Tissue distribution of ACE2 protein, the functional receptor for SARS coronavirus. A first step in understanding SARS pathogenesis. J Pathol. 2004;203(2):631‐637. 15141377 10.1002/path.1570 PMC7167720

[15] Vickers C , Hales P , Kaushik V , et al. Hydrolysis of biological peptides by human angiotensin‐converting enzyme‐related carboxypeptidase. J Biol Chem. 2002;277(17):14838‐14843. 11815627 10.1074/jbc.M200581200

[16] Zisman LS , Keller RS , Weaver B , et al. Increased angiotensin‐(1–7)‐forming activity in failing human heart ventricles – evidence for upregulation of the angiotensin‐converting enzyme homologue ACE2. Circulation. 2003;108(14):1707‐1712. 14504186 10.1161/01.CIR.0000094734.67990.99

[17] Santos RAS , Ferreira AJ , Silva ACE . Recent advances in the angiotensin‐converting enzyme 2‐angiotensin(1–7)‐Mas axis. Exp Physiol. 2008;93(5):519‐527. 18310257 10.1113/expphysiol.2008.042002

[18] Burrell LM , Risvanis J , Kubota E , et al. Myocardial infarction increases ACE2 expression in rat and humans. Eur Heart J. 2005;26(4):369‐375; discussion 322‐364. 15671045 10.1093/eurheartj/ehi114

[19] Clarke NE , Turner AJ . Angiotensin‐converting enzyme 2: the first decade. Int J Hypertens. 2012;2012:307315. 22121476 10.1155/2012/307315 PMC3216391

[20] Goulter AB , Goddard MJ , Allen JC , Clark KL . ACE2 gene expression is up‐regulated in the human failing heart. BMC Med. 2004;2(1):2–19. 15151696 10.1186/1741-7015-2-19 PMC425604

[21] Karram T , Abbasi A , Keidar S , et al. Effects of spironolactone and eprosartan on cardiac remodeling and angiotensin‐converting enzyme isoforms in rats with experimental heart failure. Am J Physiol‐Heart C. 2005;289(4):H1351‐H1358. 10.1152/ajpheart.01186.2004 15894569

[22] Gallagher PE , Ferrario CM , Tallant EA . Regulation of ACE2 in cardiac myocytes and fibroblasts. Am J Physiol Heart Circ Physiol. 2008;295(6):H2373‐H2379. 18849338 10.1152/ajpheart.00426.2008 PMC2614534

[23] Oudit GY , Kassiri Z , Jiang C , et al. SARS‐coronavirus modulation of myocardial ACE2 expression and inflammation in patients with SARS. Eur J Clin Invest. 2009;39(7):618‐625. 19453650 10.1111/j.1365-2362.2009.02153.x PMC7163766

[24] Ferrario CM , Jessup J , Chappell MC , et al. Effect of angiotensin‐converting enzyme inhibition and angiotensin II receptor blockers on cardiac angiotensin‐converting enzyme 2. Circulation. 2005;111(20):2605‐2610. 15897343 10.1161/CIRCULATIONAHA.104.510461

[25] Ishiyama Y , Gallagher PE , Averill DB , Tallant EA , Brosnihan KB , Ferrario CM . Upregulation of angiotensin‐converting enzyme 2 after myocardial infarction by blockade of angiotensin II receptors. Hypertension. 2004;43(5):970‐976. 15007027 10.1161/01.HYP.0000124667.34652.1a

[26] Gheblawi M , Wang K , Viveiros A , et al. Angiotensin‐converting enzyme 2: SARS‐CoV‐2 receptor and regulator of the renin‐angiotensin system: celebrating the 20th anniversary of the discovery of ACE2. Circ Res. 2020;126(10):1456‐1474. 32264791 10.1161/CIRCRESAHA.120.317015 PMC7188049

[27] Ciaglia E , Vecchione C , Puca AA . COVID‐19 infection and circulating ACE2 levels: protective role in women and children. Front Pediatr. 2020;8:206. 32391299 10.3389/fped.2020.00206 PMC7192005

[28] Walls AC , Park YJ , Tortorici MA , Wall A , McGuire AT , Veesler D . Structure, function, and antigenicity of the SARS‐CoV‐2 spike glycoprotein. Cell. 2020;181(2):281‐292 e286. 32155444 10.1016/j.cell.2020.02.058 PMC7102599

[29] Coutard B , Valle C , de Lamballerie X , Canard B , Seidah NG , Decroly E . The spike glycoprotein of the new coronavirus 2019‐nCoV contains a furin‐like cleavage site absent in CoV of the same clade. Antiviral Res. 2020;176:104742. 32057769 10.1016/j.antiviral.2020.104742 PMC7114094

[30] YMYHTWAPXWH: ACE2 shedding and FURIN abundance in target organs may influence the efficiency of SARS‐CoV‐2 entry. chinaXiv 2020.

[31] Braun E , Sauter D . Furin‐mediated protein processing in infectious diseases and cancer. Clin Transl Immunol. 2019;8(8):e1073. 10.1002/cti2.1073 PMC6682551 31406574

[32] Wu C , Zheng M , Yang Y . Furin: A Potential Therapeutic Target for COVID‐19. iScience. 2020;23 (10):101642. 10.1016/j.isci.2020.101642. 33043282 PMC7534598

[33] Ichiki T , Burnett JC . Post‐transcriptional modification of pro‐BNP in heart failure: Is glycosylation and circulating furin key for cardiovascular homeostasis? Eur Heart J. 2014;35(43):3001‐3003. 25246481 10.1093/eurheartj/ehu381

[34] Hoffmann M , Kleine‐Weber H , Krüger N , Müller M , Drosten C , Pöhlmann S . The novel coronavirus 2019 (2019‐nCoV) uses the SARS‐coronavirus receptor ACE2 and the cellular protease TMPRSS2 for entry into target cells. bioRxiv: the preprint server for biology 2020:2020.2001.2031.929042.

[35] Lukassen S , Chua RL , Trefzer T , et al. SARS‐CoV‐2 receptor ACE2 and TMPRSS2 are primarily expressed in bronchial transient secretory cells. EMBO J. 2020;39(10):e105114 32246845 10.15252/embj.20105114 PMC7232010

[36] Lambert DW , Yarski M , Warner FJ , et al. Tumor necrosis factor‐alpha convertase (ADAM17) mediates regulated ectodomain shedding of the severe‐acute respiratory syndrome‐coronavirus (SARS‐CoV) receptor, angiotensin‐converting enzyme‐2 (ACE2). J Biol Chem. 2005;280(34):30113‐30119. 15983030 10.1074/jbc.M505111200 PMC8062222

[37] Abassi Z , Goltsman I , Karram T , Winaver J , Hoffman A . Aortocaval fistula in rat: a unique model of volume‐overload congestive heart failure and cardiac hypertrophy. J Biomed Biotechnol. 2011;2011:729497. 21274403 10.1155/2011/729497 PMC3025398

[38] Huentelman MJ , Grobe JL , Vazquez J , et al. Protection from angiotensin II‐induced cardiac hypertrophy and fibrosis by systemic lentiviral delivery of ACE2 in rats. Exp Physiol. 2005;90(5):783‐790. 16049057 10.1113/expphysiol.2005.031096

[39] Diez‐Freire C , Vazquez J , de Adjounian MFC , et al. ACE2 gene transfer attenuates hypertension‐linked pathophysiological changes in the SHR. Physiol Genomics. 2006;27(1):12‐19. 16788004 10.1152/physiolgenomics.00312.2005

[40] Grobe JL , Der Sarkissian S , Stewart JM , Meszaros JG , Raizada MK , Katovich MJ . ACE2 overexpression inhibits hypoxia‐induced collagen production by cardiac fibroblasts. Clin Sci. 2007;113(7–8):357‐364. 10.1042/CS20070160 17600530

[41] Muniyappa R , Gubbi S . COVID‐19 pandemic, coronaviruses, and diabetes mellitus. Am J Physiol Endocrinol Metab. 2020;318(5):E736‐E741. 32228322 10.1152/ajpendo.00124.2020 PMC7191633

[42] Iwai M , Horiuchi M . Devil and angel in the renin‐angiotensin system: ACE‐angiotensin II‐AT1 receptor axis vs. ACE2‐angiotensin‐(1–7)‐Mas receptor axis. Hypertens Res. 2009;32(7):533‐536. 19461648 10.1038/hr.2009.74 PMC7091931

[43] Crackower MA , Sarao R , Oudit GY , et al. Angiotensin‐converting enzyme 2 is an essential regulator of heart function. Nature. 2002;417(6891):822‐828. 12075344 10.1038/nature00786

[44] Yamamoto K , Ohishi M , Katsuya T , et al. Deletion of angiotensin‐converting enzyme 2 accelerates pressure overload‐induced cardiac dysfunction by increasing local angiotensin II. Hypertension. 2006;47(4):718‐726. 16505206 10.1161/01.HYP.0000205833.89478.5b

[45] Danilczyk U , Penninger JM . Angiotensin‐converting enzyme II in the heart and the kidney. Circ Res. 2006;98(4):463‐471. 16514079 10.1161/01.RES.0000205761.22353.5f

[46] Kassiri Z , Zhong JC , Guo D , et al. Loss of angiotensin‐converting enzyme 2 accelerates maladaptive left ventricular remodeling in response to myocardial infarction. Circ‐Heart Fail. 2009;2(5):446‐455. 19808375 10.1161/CIRCHEARTFAILURE.108.840124

[47] Pieruzzi F , Abassi ZA , Keiser HR . Expression of renin‐angiotensin system components in the heart, kidneys, and lungs of rats with experimental heart‐failure. Circulation. 1995;92(10):3105‐3112. 7586282 10.1161/01.cir.92.10.3105

[48] Schroten NF , Gaillard CAJM , van Veldhuisen DJ , Szymanski MK , Hillege HL , de Boer RA . New roles for renin and prorenin in heart failure and cardiorenal crosstalk. Heart Fail Rev. 2012;17(2):191‐201. 21695549 10.1007/s10741-011-9262-2 PMC3310995

[49] Schrier RW , Abraham WT . Mechanisms of disease – hormones and hemodynamics in heart failure. N Engl J Med. 1999;341(8):577‐585. 10451464 10.1056/NEJM199908193410806

[50] Katz AM . Heart failure: a hemodynamic disorder complicated by maladaptive proliferative responses. J Cell Mol Med. 2003;7(1):1‐10. 12767256 10.1111/j.1582-4934.2003.tb00197.x PMC6740240

[51] Braunwald E . Heart failure. JACC Heart Failure. 2013;1(1):1‐20. 24621794 10.1016/j.jchf.2012.10.002

[52] Gheblawi M , Wang KM , Viveiros A , et al. Angiotensin‐converting enzyme 2: SARS‐CoV‐2 receptor and regulator of the renin‐angiotensin system celebrating the 20th anniversary of the discovery of ACE2. Circ Res. 2020;126(10):1456‐1474. 32264791 10.1161/CIRCRESAHA.120.317015 PMC7188049

[53] Li WH , Moore MJ , Vasilieva N , et al. Angiotensin‐converting enzyme 2 is a functional receptor for the SARS coronavirus. Nature. 2003;426(6965):450‐454. 14647384 10.1038/nature02145 PMC7095016

[54] Letko M , Marzi A , Munster V . Functional assessment of cell entry and receptor usage for SARS‐CoV‐2 and other lineage B betacoronaviruses. Nat Microbiol. 2020;5(4):562–569. 32094589 10.1038/s41564-020-0688-y PMC7095430

[55] Wan YS , Shang J , Graham R , Baric RS , Li F . Receptor recognition by the novel coronavirus from Wuhan: an analysis based on decade‐long structural studies of SARS coronavirus. J Virol. 2020;94(7):e00127‐20. 31996437 10.1128/JVI.00127-20 PMC7081895

[56] Khan S , Chen L , Yang C‐R , Raghuram V , Khundmiri SJ , Knepper MA . Does SARS‐CoV‐2 infect the kidney? J Am Soc Nephrol. 2020;31(12):2746‐2748. 33051359 10.1681/ASN.2020081229 PMC7790203

[57] Perico L , Benigni A , Remuzzi G . Should COVID‐19 concern nephrologists? Why and to what extent? The emerging impasse of angiotensin blockade. Nephron. 2020;144(5):213‐221. 32203970 10.1159/000507305 PMC7179544

[58] Zheng YY , Ma YT , Zhang JY , Xie X . COVID‐19 and the cardiovascular system. Nat Rev Cardiol. 2020;17(5):259‐260. 32139904 10.1038/s41569-020-0360-5 PMC7095524

[59] Basso C , Leone O , Rizzo S , et al. Pathological features of COVID‐19‐associated myocardial injury: a multicentre cardiovascular pathology study. Eur Heart J. 2020;41(39):3827‐3835. 32968776 10.1093/eurheartj/ehaa664 PMC7543528

[60] Shovlin CL , Vizcaychipi MP . Vascular inflammation and endothelial injury in SARS‐CoV‐2 infection: the overlooked regulatory cascades implicated by the ACE2 gene cluster. QJM Int J Med. 2020;1–6. 10.1093/qjmed/hcaa241 PMC7454888 32777054

[61] Santos RA . Angiotensin‐(1–7). Hypertension. 2014;63(6):1138‐1147. 24664288 10.1161/HYPERTENSIONAHA.113.01274

[62] Cui D , Liu Y , Jiang X , et al. Single‐cell RNA expression profiling of ACE2 and TMPRSS2 in the human trophectoderm and placenta. Ultrasound Obstet Gynecol 2020;57(2):256–284. 10.1002/uog.22186 PMC7461088 32851697

[63] Ichiki T , Huntley BK , Burnett JC Jr . BNP molecular forms and processing by the cardiac serine protease corin. Adv Clin Chem. 2013;61:1‐31. 24015598 10.1016/b978-0-12-407680-8.00001-4 PMC4522930

