# Supplementary figures and images for: Pulmonary, cardiac and renal distribution of ACE2, furin, TMPRSS2 and ADAM17 in rats with heart failure: Potential implication for COVID‐19 disease
Source: J Cell Mol Med. 2021 Mar 4;25(8):3840–55. doi: 10.1111/jcmm.16310 (PMC8014258; doi:10.1111/jcmm.16310)

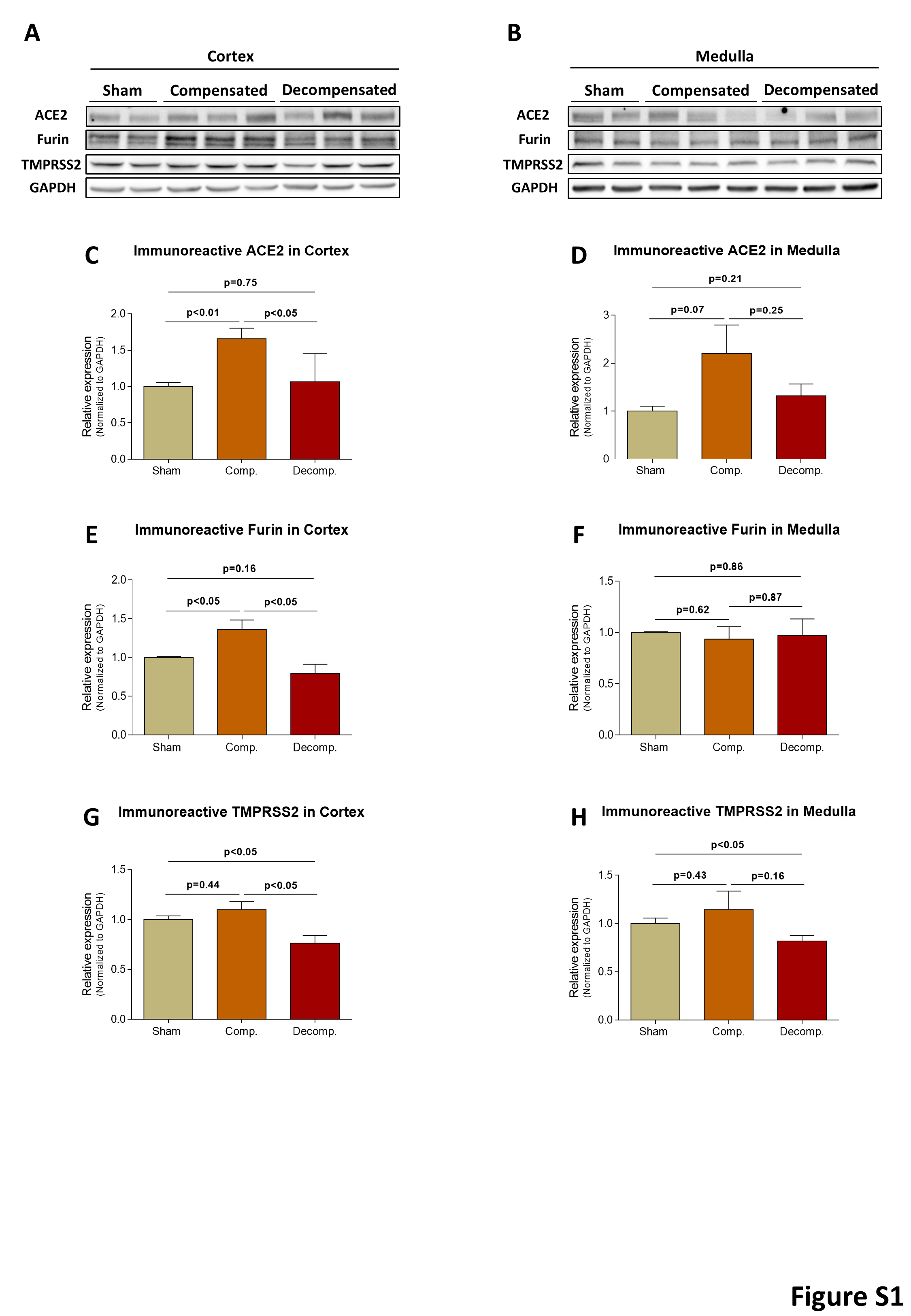

Supplement: Supplementary file 1 — Fig S1 [file JCMM-25-3840-s001.TIF]

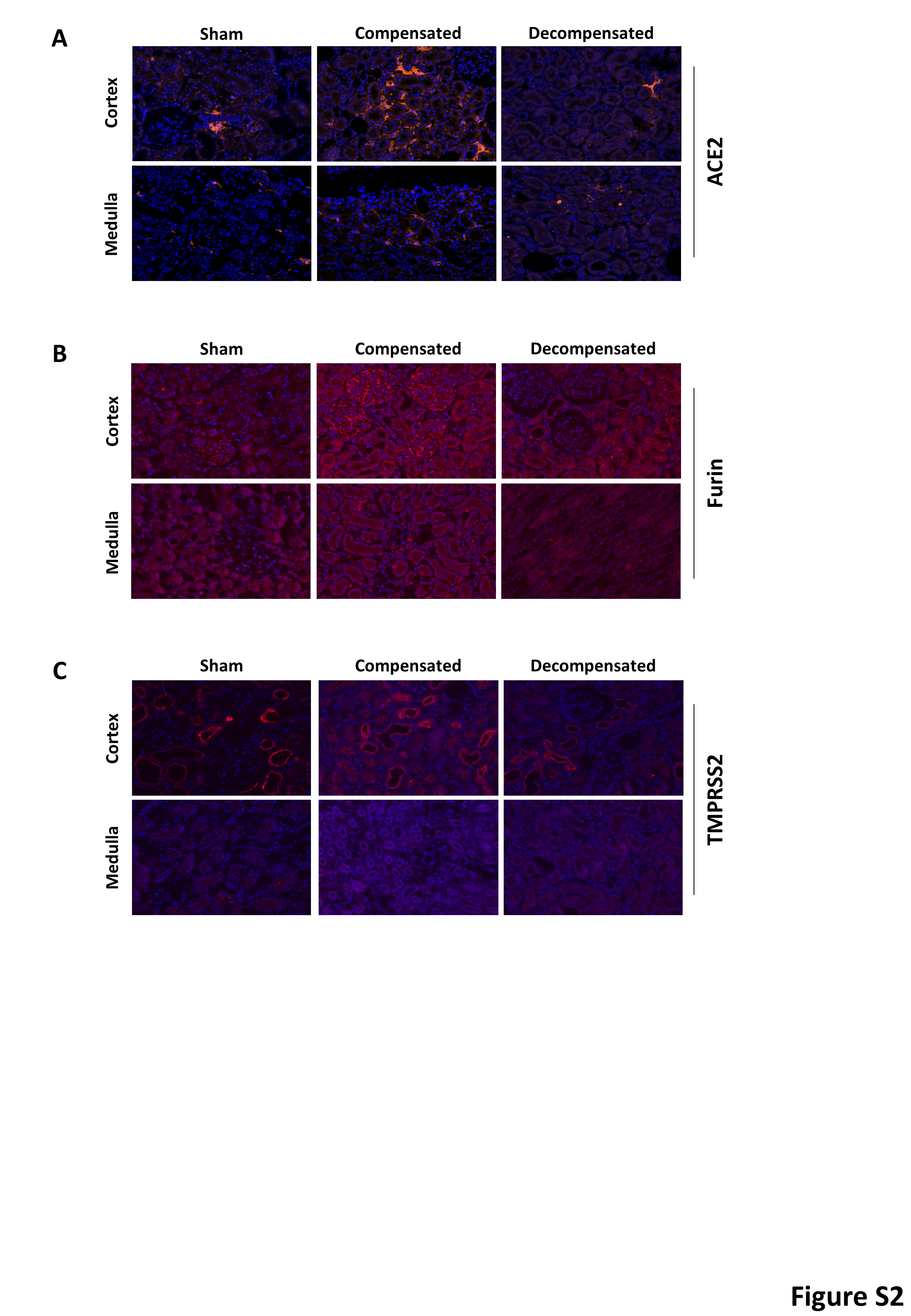

Supplement: Supplementary file 2 — Fig S2 [file JCMM-25-3840-s002.TIF]
